# Profiling Serum Oxylipin Metabolites Across Melanoma Subtypes and Immunotherapy Responders

**DOI:** 10.3390/metabo16010014

**Published:** 2025-12-23

**Authors:** Alexander C. Goodman, Kylie M. Michel, Morgan L. MacBeth, Jaqueline A. Turner, Richard P. Tobin, William A. Robinson, Kasey L. Couts

**Affiliations:** 1 Department of Medicine, Division of Medical Oncology, University of Colorado Anschutz Medical Campus, Aurora, CO 80045, USA; 2 College of Osteopathic Medicine, Rocky Vista University, Englewood, CO 80112, USA; 3 Department of Surgery, University of Colorado Anschutz Medical Campus, Aurora, CO 80045, USA

**Keywords:** oxylipins, melanoma, rare melanomas, immunotherapy, prostaglandin J2

## Abstract

Background/Objectives: Immunotherapy has significantly improved clinical outcomes for patients with late-stage melanoma, yet a substantial portion of patients fail to respond to these treatments. The variability in responses to immunotherapy, both among individual patients and across different melanoma subtypes, underscores the need to explore the influence of circulating factors such as oxylipins on therapeutic outcomes. This study investigated the relationship between serum oxylipin profiles and response to immune checkpoint inhibitor therapy in melanoma subtypes to identify potential metabolic biomarkers for treatment response. Methods: In a retrospective cohort study, serum samples from 43 stage III and stage IV melanoma patients treated at the University of Colorado Hospital from 2010 to 2023 were analyzed via ultra-high-pressure liquid chromatography-mass spectrometry. Melanoma patients were treated with anti-PD-1 monotherapy or combination immune checkpoint inhibitor therapy, and response was assessed using RECIST 1.1 criteria. Results: We determined that global oxylipin metabolite profiles are largely uniform pre- and post-treatment across melanoma subtypes, including cutaneous, acral, mucosal, and uveal melanoma. Prostaglandin J_2_ was more abundant in rare melanoma subtypes, including acral, mucosal, and uveal melanoma, compared to cutaneous melanoma. Conclusions: Despite limited variation in serum oxylipin molecular species by subtype and response status, we observed significant differences in prostaglandin J_2_, which could serve as a potential biomarker for immune checkpoint inhibitor therapy response in melanoma.

## 1. Introduction

Oxylipins are a family of fatty acids that have undergone at least one oxidation reaction and are typically derived from polyunsaturated fatty acids (PUFAs) like omega-3 and omega-6 fatty acids. These molecules are produced through three primary pathways: cyclooxygenases (COXs), lipoxygenases (LOXs), and cytochrome P450 enzymes (CYPs) [1]. Common omega-3 PUFAs, such as eicosapentaenoic acid (EPA) and docosahexaenoic acid (DHA), give rise to derivatives like hydroxyeicosapentaenoic acids (HEPEs), hydroxydocosahexaenoic acids (HDoHEs), and resolvin D1 (RvD1), which are pro-resolving and anti-inflammatory lipid mediators [2]. In contrast, omega-6 PUFAs, such as arachidonic acid (AA), produce metabolites like prostaglandins (PGs), thromboxanes (TXs), leukotrienes (LTs), and hydroxyeicosatetraenoic acids (HETEs), which are pro-inflammatory lipids [2]. Additional PUFAs, including dihomo-gamma-linolenic acid (DGLA) and linolenic acid (LA), also produce oxylipin derivatives that display both pro- and anti-inflammatory activities, further complicating the role of these molecules in inflammatory processes [1].

Previous reports show oxylipin molecular species, specifically prostaglandin E_2_ (PGE_2_), are elevated in melanoma. PGE_2_ is generated from COX-2, which has elevated expression in melanoma and is associated with increased proliferation, enhanced invasion, and a worse overall prognosis [3,4]. Additionally, the suppression of COX-2 leads to enhanced infiltration of CD8^+^ T cells and dendritic cells into the tumor microenvironment, suggesting COX-2 modulates adaptive immune responses [5]. PGE_2_ can significantly influence the efficacy of immune checkpoint inhibitor (ICI) therapy in cancers, including melanoma. Elevated tumoral PGE_2_ levels are associated with immunotherapy resistance [6,7,8], and inhibition of COX rescues anti-PD-1 efficacy in resistant tumors [6]. Combination anti-CTLA-4 antibodies and prostaglandin E_2_ receptor 4 (EP4) antagonists, which block PGE_2_ signaling, enhances tumor cytoreduction [7]. Even omega-3 fatty acids enhance ICI response, whereas omega-6 fatty acids impede efficacy. Higher plasma levels of long-chain fatty acids, especially omega-3s, are associated with better ICI responses in melanoma patients [9], suggesting that circulating lipid profiles may influence immunotherapy efficacy and serve as potential biomarkers for predicting patient response and potential reprogramming of inflammatory and adaptive immune responses during ICI therapy.

While prostaglandins have been widely studied in melanoma, the contributions of other oxylipins, including leukotrienes and LOX-derived metabolites, remain less understood [4]. In vitro melanoma treatment with LOX inhibition, specifically 15-LOX inhibitors, results in reduced proliferation, decreased cell viability, and enhanced cytotoxicity compared to COX inhibitors [10]. Interestingly, melanoma-reprogrammed Schwann cells have been found to upregulate 12-LOX, 15-LOX, and COX-2, leading to increased production of pro-tumorigenic oxylipins, including PGE_2_ and lipoxins A_4_/B_4_, and subsequent anti-tumor T-cell activation [11]. These findings suggest that both COX and LOX pathways are instrumental in melanoma progression and immune evasion.

Rarer subtypes of melanoma include acral melanoma (AM), occurring on the palms, soles, fingers, toes, and nail units; mucosal melanoma (MM), which arises from mucosal surfaces; and uveal melanoma (UM), originating from melanocytes in the iris, ciliary body, and choroid of the eye [12]. Existing studies indicate that these rare melanomas exhibit lower response rates to ICI therapy. For instance, patients with acral melanoma show an objective response rate of 32% to combination ICI therapy, which is lower than that observed in cutaneous melanoma [13]. Mucosal melanoma responds less effectively to both single-agent and combination ICI therapy, with a progression-free survival of 5.9 months compared to 11.7 months in cutaneous melanoma patients receiving combination therapy [14]. Uveal melanoma patients demonstrate an even lower objective response rate of 18% to combination ICI therapy [13]. While genomic and transcriptomic differences among these melanoma subtypes have been identified [15,16,17], research has predominantly focused on these genetic variations, with little attention given to metabolomics, particularly lipidomics. Notably, a study by Vilbert and colleagues (2023) demonstrated that cutaneous melanoma (CM), MM, and UM exhibit distinct metabolic profiles, specifically in lipid composition [12]. They analyzed choline-containing phospholipids and sphingolipids, finding that serum levels of these lipids were lower in patients with MM and higher in those with CM and UM [12]. Similarly, de Bruyn et al. (2023) found significant differences in the metabolome of UM patients compared to healthy controls, particularly in metabolites associated with malignant processes [18]. These studies suggest that metabolic differences, especially in lipid profiles, exist among melanoma subtypes and may contribute to their distinct biological behaviors. Given the diverse responses to ICI therapy among melanoma subtypes and the potential influence of oxylipins on therapeutic outcomes, there is a critical need to understand how circulating oxylipin profiles correlate with ICI responses across different melanoma types. Peripheral blood analysis offers a less invasive and more accessible means to identify biomarkers predictive of treatment response, circumventing the challenges associated with tumor biopsies. This study investigates the association between circulating levels of oxylipins and the response to immune checkpoint blockade in patients with different melanoma subtypes. By profiling oxylipins in peripheral blood, we seek to identify metabolic signatures that correlate with ICI therapy outcomes. Specifically, differences in pro-inflammatory and anti-inflammatory oxylipins may provide insights into the mechanisms of ICI resistance and predict therapeutic efficacy as potential biomarkers for personalized treatment strategies.

## 2. Materials and Methods

### 2.1. Study Population

This is an exploratory retrospective observational cohort study of patients with advanced melanoma treated with ICI therapy at the University of Colorado Hospital. Serum samples were collected between 2010 and 2023. Samples were collected in red-top tubes directly in the hospital, processed immediately, and stored in a −80 °C freezer until use. For the purposes of our study, we focused on the PD-1 immune checkpoint blockers. Patients were included in the study if they received either pembrolizumab (Keytruda) or nivolumab (Opdivo) monotherapy or combination therapy [nivolumab (Opdivo) + ipilimumab (Yervoy)]. When possible, we collected a pre-treatment ICI therapy serum sample and an on-treatment ICI therapy sample at the time of their first follow-up. If serum samples were drawn more than 6 months prior to treatment start, they were excluded from treatment-related analysis.

Response was defined according to the RECIST 1.1 criteria [19]. We classified a patient as a responder if they showed a complete response (CR) or partial response (PR), and non-responders were designated as stable disease (SD) or progressive disease (PD) at the time of follow-up. Follow-up time averaged around one year and ranged from zero to six years.

### 2.2. Oxylipin Quantification by UHPLC-MS

Oxylipin profiles were measured in human melanoma serum samples using mass spectrometry. Serum samples were diluted 1:10 with cold 5:3:2 MeOH:ACN:H_2_O (*v*/*v*/*v*) containing deuterated standards (Cayman Chemical, Ann Arbor, USA), each at a final concentration of 6 ng/μL (Supplementary Table S1). Samples were vortexed vigorously for 30 min at 4 °C, then centrifuged for 10 min at 18,213 RCF. Using 10 uL injection volumes, the supernatants were analyzed by ultra-high-pressure liquid chromatography coupled to mass spectrometry (UHPLC-MS). Metabolites were resolved across a 1.7 μm, 2.1 × 100 mm Waters Acquity BEH column using a 7 min gradient previously described [20].

Oxylipin quantification was performed using a stable-isotope dilution (SID) approach with exogenously added deuterated internal standards to compensate for extraction efficiency, matrix effects, and ionization variability. Internal standards were added to all samples prior to extraction and used for absolute quantification by peak area ratio comparison. Data were acquired in an unbiased, high-resolution MS1 scanning mode across a broad mass range, enabling both quantitative targeted analysis and post hoc interrogation of the broader oxylipin lipidome.

Quality control included solvent blanks to assess background signal and pooled technical QC samples generated by combining aliquots from all study samples. QC samples were injected at the beginning, middle, and end of each run sequence to monitor instrument stability and analytical drift. Retention time stability and coefficient of variation (CV) for detected oxylipins were assessed across QC injections to ensure data robustness. [20,21].

Following data acquisition, .raw files were converted to .mzXML using the RawConverter application. Metabolites were annotated and peaks integrated based on intact mass, 13C isotope pattern, and retention times in conjunction with the deuterated standards and KEGG database. Peaks were integrated using El-Maven (Elucidata, New Dehli, India).

### 2.3. Experimental Data and Statistical Analysis

Absolute quantification of study samples was reported. All study samples were normalized to internal standards. All statistical analyses and visualizations were conducted in R (R version 4.4.0, http://www.r-project.org, URL accessed on 4 February 2024). The significance value was set to alpha = 0.05. Summaries for demographics are presented as N (%) for categorical variables and mean (SD) with range for continuous variables. Before any analysis was conducted, testing for a normal distribution of the data was conducted using the Shapiro–Wilk test of normality. Given that most measured study samples were not normally distributed in all comparisons (Supplementary Data S1), we elected to use non-parametric testing. For each metabolite, statistical significance was assessed using Kruskal–Wallis testing followed by Dunn’s post hoc correction for multiple group comparisons. However, global false discovery rate (FDR) correction across all 33 metabolites was not applied due to the exploratory design, limited cohort size, and high risk of Type II error. Accordingly, statistically significant metabolite associations are interpreted as hypothesis-generating and require independent validation.

## 3. Results

### 3.1. Patient Clinical Characteristics

Forty-three melanoma patients with at least stage III disease were included within the study cohort (Table 1) and represented the four major subtypes of melanoma, including cutaneous (*n* = 6), acral (*n* = 7), mucosal (*n* = 23), and uveal (*n* = 7). Mucosal melanoma was further subdivided into three anatomic locations: anorectal (*n* = 8), nasopharyngeal (*n* = 8), and vulvovaginal (*n* = 7). A total of 26 female and 17 male melanoma patients, ranging from 39 to 92 years old, with an average age of 63 years old, represented the entire cohort. The frequency of patients receiving anti-PD-1 immunotherapy or combination therapy (anti-PD-1 and anti-CTLA4) ranged from 14.3% to 62.5% across melanoma subtypes. Rates of ICI responders ranged from 0% (AM) to 66.7% (CM).

### 3.2. Mass Spectrometry Evaluation of Oxylipin Levels in Patient Serum

A total of 33 oxylipin metabolites were analyzed using mass spectrometry (Supplementary Table S1). Each of the lipid metabolites was further classified into six polyunsaturated fatty acids (PUFAs), 15 arachidonic acid (AA) derivatives, four docosahexaenoic acid (DHA) derivatives, one eicosapentaenoic acid (EPA) derivative, and seven linoleic acid (LA) derivatives (Supplementary Figure S1). Although dihomo-γ-linolenic acid (DGLA) is displayed within the LA-derived metabolite grouping in the PCA loading plots.

Based on its biosynthetic origin from linoleic acid, we note that DGLA is metabolically intermediate between the LA and arachidonic acid (AA) pathways. DGLA can contribute to both series-1 prostaglandins and downstream AA-associated lipid mediators; therefore, its classification here reflects precursor relationships rather than exclusive pathway membership. We performed an unsupervised principal component analysis (PCA) for all samples and identified four outlier samples, which were removed from further analysis (Supplementary Figure S2a). A heatmap generated from unsupervised clustering of all specimens showed polyunsaturated fatty acids clustered separately from other fatty acids, as expected (Supplementary Figure S2b).

### 3.3. Baseline Serum Oxylipins Across Melanoma Subtypes

**Figure 1:**
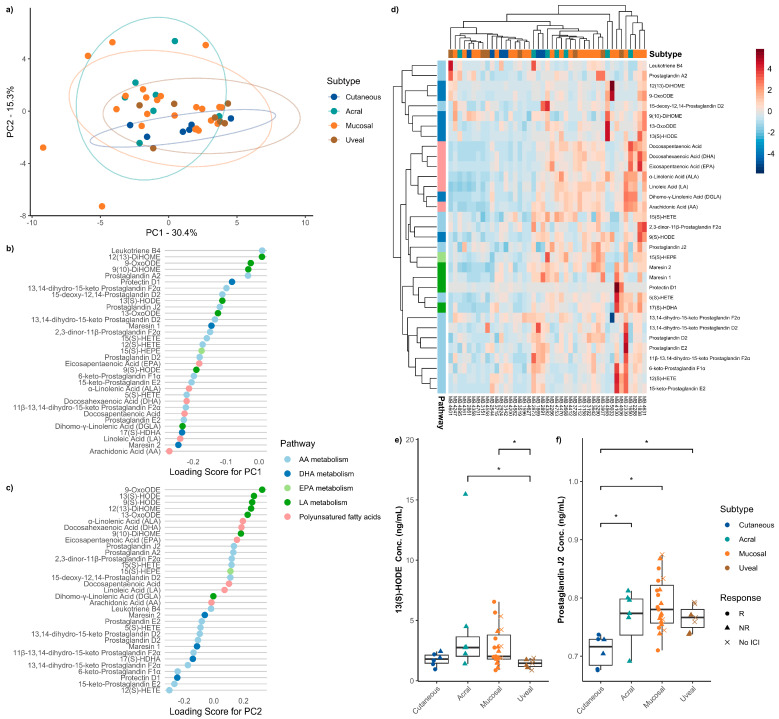
Summary of comparison between melanoma subtypes. (**a**) Principal component analysis discriminating against healthy donors and melanoma subtypes. The explained variance of each component is indicated on the corresponding axis. Plot of loading scores for (**b**) PC1 and (**c**) PC2 of the principal component analysis. Plotted values are absolute to signify the magnitude of effect each oxylipin has on the principal component. (**d**) Heatmap demonstrating differences in serum oxylipin concentration between melanoma subtypes. Boxplot of absolute serum concentration of (**e**) 13(S)-HODE and (**f**) prostaglandin J_2_ for all melanoma subtypes before any ICI therapy. Significant differences are indicated by an asterisk (*p* ≤ 0.05).

To evaluate which oxylipins differed between the melanoma subtypes at baseline, oxylipin concentrations were analyzed in ICI therapy-naïve patient serum samples. First, we performed an unsupervised PCA. The first two principal components, PC1 and PC2, accounted for greater than 45% of the variation in the data and demonstrated a significant amount of overlap among melanoma subtypes (Figure 1a). We calculated the loading scores for the first and second principal components to determine which lipids contributed the most variation. Assessment of the top 10 oxylipins with the highest loading scores showed that a significant portion of these oxylipins were part of LA metabolism (Figure 1b,c). Unsupervised hierarchical clustering revealed no distinct global clustering pattern based on melanoma subtype (Figure 1d), which was further supported by post hoc analysis using the Kruskal–Wallis rank sum test with Dunn’s test (Supplementary Data S2).

Upon further stratifying the oxylipins, we did find that 13(S)-HODE was enriched in acral and mucosal melanoma compared to uveal melanoma (*H* = 9.584, *p* = 0.022). Prostaglandin J_2_ (PGJ_2_) was significantly more abundant in rare melanomas, including acral, mucosal, and uveal, as compared to cutaneous melanoma (H = 13.715, *p* = 0.003). Both acral (*p* = 0.028) and mucosal (*p* = 0.028) melanoma patients had significantly higher serum levels of 13(S)-HODE than uveal melanoma (Figure 1e). PGJ2 was higher in acral (*p* = 0.048), mucosal (*p* = 0.001), and uveal (*p* = 0.050) melanoma patients when compared to cutaneous (Figure 1f). There was a notable trend towards decreased 11β-13,14-dihydro-15-keto prostaglandin F2α in rare melanomas compared to cutaneous (H = 6.378, *p* = 0.095) and enrichment in 13-OxoODE in acral and mucosal melanoma (H = 6.667, *p* = 0.083) (Supplementary Figure S3. While not reaching significance, cutaneous (*p* = 0.131), acral (*p* = 0.129), and mucosal (*p* = 0.175) melanoma patients had higher levels of serum 11β-13,14-dihydro-15-keto prostaglandin F2α at baseline than uveal melanoma patients. Similarly, there were no significant differences in serum 13-OxoODE across the melanoma subtypes, but acral (*p* = 0.111) and mucosal (*p* = 0.111) patients showed higher levels than uveal patients. Together, these results suggest that while there are global profile differences in oxylipin metabolism across subtypes, AA and LA metabolite derivatives and 13(S)-HODE and PGJ2 are differentially enriched in rare melanoma subtypes.

### 3.4. Baseline Serum Oxylipins Across Mucosal Melanoma Anatomic Locations

**Figure 2:**
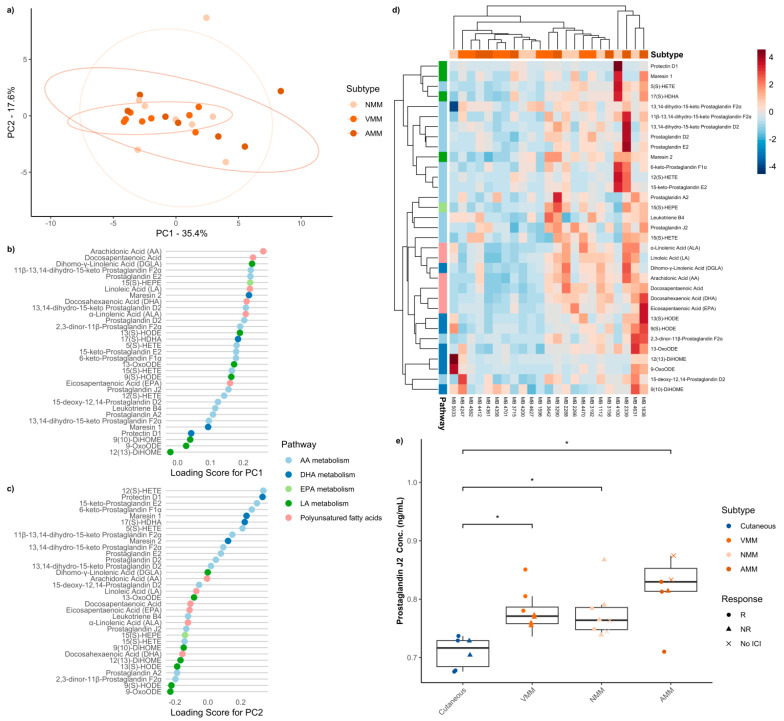
Summary of comparison between mucosal melanoma anatomic locations. (**a**) Principal component analysis discriminating against mucosal melanoma locations. The explained variance of each component is indicated on the corresponding axis. Plot of loading scores for (**b**) PC1 and (**c**) PC2 of the principal component analysis. Plotted values are absolute to signify the magnitude of effect each oxylipin has on the principal component. (**d**) Heatmap demonstrating differences in serum oxylipin concentration between mucosal melanoma locations. (**e**) Boxplot of absolute serum concentration of prostaglandin J_2_ for cutaneous melanoma and mucosal melanoma locations before any ICI therapy. Significant differences are indicated by an asterisk (*p* ≤ 0.05).

Next, we questioned whether circulating oxylipin abundance differed across mucosal melanoma anatomic locations. ICI therapy-naïve samples from patients with anorectal, nasopharyngeal, and vulvovaginal mucosal melanoma were compared, and two-dimensional PCA accounted for greater than 53% of the variation in the data, with clustered sample overlap despite different mucosal etiologies (Figure 2a). Assessment of the top 10 oxylipins with the highest loading scores showed that a significant portion of these oxylipins were part of the PUFAs (Figure 2b,c). Hierarchical clustering analysis did not delineate distinct metabolic profiles despite different anatomic locations (Figure 2d). Further analysis on individual metabolites recapitulated an enriched abundance of PGJ_2_ in mucosal melanomas compared to cutaneous, but no significant difference was observed across mucosal anatomic location (*H* = 14.854, *p* = 0.002) (Figure 2e). No other individual metabolites showed any notable enrichments (Supplementary Data S3, Supplementary Figure S4). Cutaneous melanoma patients showed significantly lower baseline serum levels of PGJ_2_ than vulvovaginal (*p* = 0.025) and nasopharyngeal (*p* = 0.025) mucosal melanoma patients, with anorectal (*p* = 0.001) mucosal melanoma patients having the highest levels and most statistically different from cutaneous (Figure 2e). Thus, oxylipin metabolism shows variation in the AA metabolic pathway with increased abundance of PGJ_2_ across all mucosal melanoma anatomic primary sites.

### 3.5. Baseline Serum Oxylipins Between Immune Checkpoint Therapy Responders and Non-Responders Across Melanoma Subtypes

**Figure 3:**
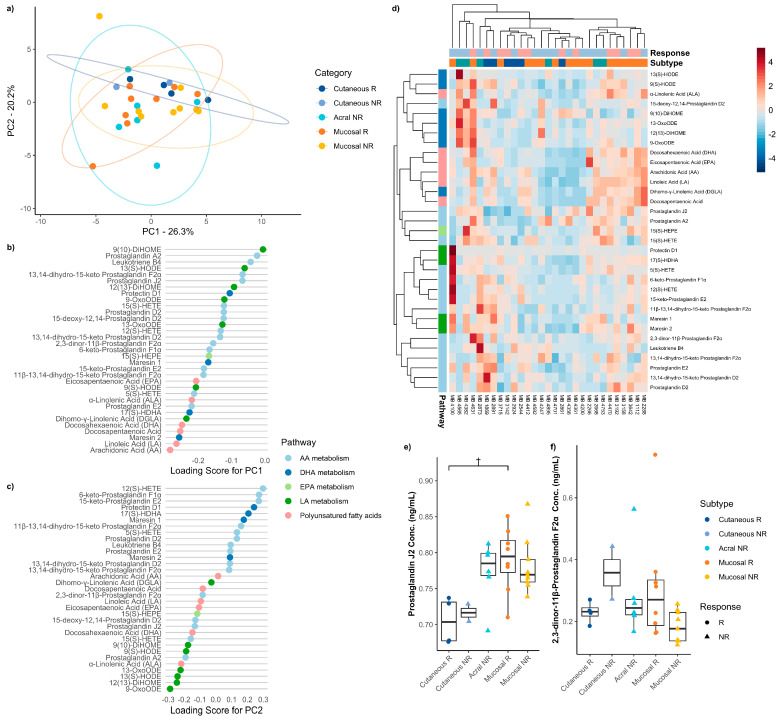
Summary of comparison between ICI therapy responders and non-responders for each melanoma subtype. (**a**) Principal component analysis discriminating against ICI therapy responders and non-responders across melanoma subtypes. The explained variance of each component is indicated on the corresponding axis. Plot of loading scores for (**b**) PC1 and (**c**) PC2 of the principal component analysis. Plotted values are absolute to signify the magnitude of effect each oxylipin has on the principal component. (**d**) Heatmap demonstrating differences in serum oxylipin concentration between ICI therapy responders and non-responders across melanoma subtypes. Boxplot of absolute serum concentration of (**e**) prostaglandin J_2_ and (**f**) 2,3-dinor-11β-prostaglandin F_2α_ for ICI therapy responders and non-responders across melanoma subtypes before any ICI therapy. Acral responders were not included in these plots as there were no acral responders in our study population. Trend differences are indicated by a cross (*p* ≤ 0.100).

Next, we looked at the differences in baseline, pre-treatment serum oxylipins between ICI therapy responders (R) and non-responders (NR) across the four melanoma subtypes. Serum samples were divided by subtype and ICI response and compared using PCA. Samples from patients who did not go on to receive ICI therapy were excluded from the analysis. Dimension reduction analysis demonstrated overlap of individual patient samples and overall uniform distribution of the data variation despite subtype and response status, where two-dimensional PCA accounted for greater than 46% of the variation (Figure 3a). Assessment of the top 10 oxylipins with the highest loading scores showed that components were comprised of AA, DHA, and LA metabolites (Figure 3b,c). Unsupervised hierarchical clustering did not distinguish response status based on global metabolic profile (Figure 3d); however, some mucosal samples clustered based on enrichment of PUFAs.

Individual metabolite analysis showed a significantly increased abundance of PGJ_2_ in both R and NR rare melanomas (*H* = 12.066, *p* = 0.034). Specifically, cutaneous melanoma patients who responded to immune checkpoint therapy showed a trend toward lower levels of baseline serum PGJ_2_ than mucosal responders (*p* = 0.065) (Figure 3e). While not statistically significant, 2,3-dinor-11β-prostaglandin F_2α_ was trending to be higher in cutaneous NR patient serum samples (*H* = 9.699, *p* = 0.084). There were no significant or trend differences for 2,3-dinor-11β-prostaglandin F2α (Figure 3f) in the post hoc analysis. No other individual metabolites showed any notable enrichments (Supplementary Data S4, Supplementary Figure S5). These findings support the previous findings that while there are no significant global metabolic profile changes, individual metabolites distinguish rare melanoma subtypes compared to cutaneous melanomas.

### 3.6. Difference Between Pre-Treatment and on-/Post-Treatment Oxylipins Across Melanoma Subtypes

**Figure 4:**
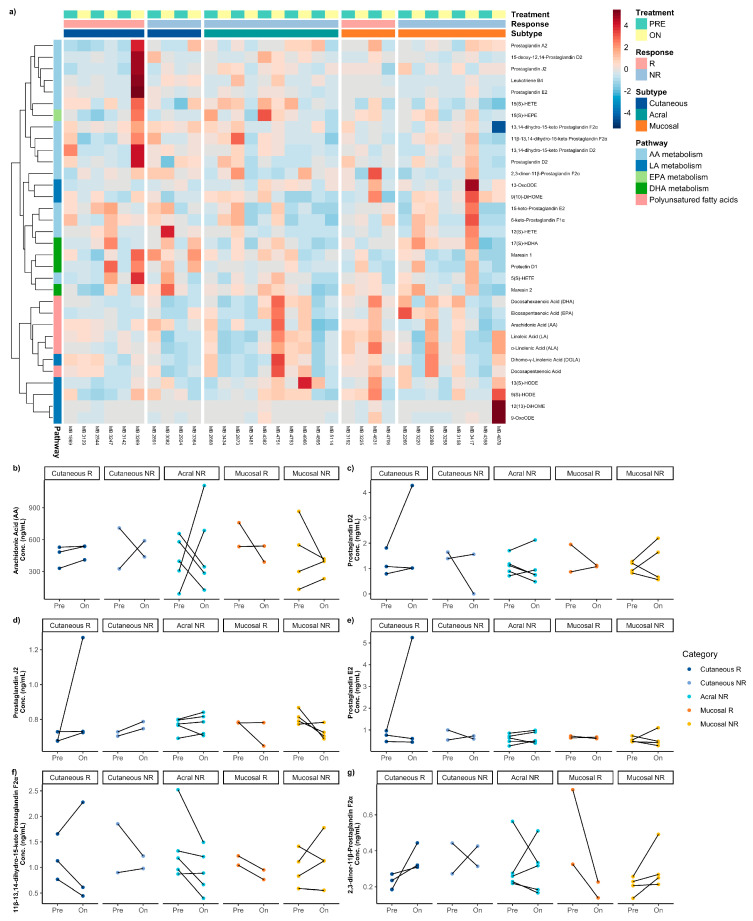
Summary of comparison between matched pre- and on-/post-treatment samples in ICI therapy responders and non-responders for each melanoma subtype. (**a**) Heatmap demonstrating differences in matched pre- and on-/post-treatment serum oxylipin concentrations between ICI therapy responders and non-responders across melanoma subtypes. Line graphs of arachidonic acid-related metabolism oxylipins for matched pre- and on-/post-treatment samples, absolute serum concentration of (**b**) arachidonic acid, (**c**) prostaglandin D_2_, (**d**) prostaglandin J_2_, (**e**) prostaglandin E2, (**f**) 11β-13,14-dihydro-15-keto prostaglandin F_2α_, and (**g**) 2,3-dinor-11β-prostaglandin F_2α_ for sample or response.

Lastly, we questioned how serum oxylipin levels change from pre-treatment ICI therapy to on-treatment or post-treatment ICI therapy across the four melanoma subtypes. Uveal melanoma subtypes were excluded in this analysis since on-/post-treatment samples were not available. Additionally, acral responders were not included in this analysis; there was only one matched sample for this group. Pathway hierarchical clustering demonstrated clustering according to the oxylipin pathway, particularly for AA, LA, PUFA, and DHA metabolites (Figure 4a). However, no consistent patterns were discerned between pre- and on-/post-treatment samples according to ICI therapy responders and non-responders across melanoma subtypes before any ICI therapy. The “On” column comprised on-treatment and post-treatment samples.

We used a paired Wilcoxon signed-rank test to test for a differences in pre-treatment and on-/post-treatment serum samples across responders and non-responders for all melanoma types (Supplementary Data S5). Given that AA and LA metabolism drove most of the variability, we assessed cancer-associated oxylipins in the AA pathway (Figure 4b–f) and the LA pathway (Supplementary Figure S6). We did not find any significant or trend differences between pre-treatment and on-/post-treatment serum samples across all the measured oxylipins for any of the melanoma types.

## 4. Discussion

While ICI therapy has revolutionized the treatment of melanoma and other cancers, a large portion of patients fail to respond to immunotherapy. Here, we investigated whether oxylipin profiles in serum samples of melanoma patients for the major melanoma subtypes correlated with response to ICI therapy. We observed limited variation in global oxylipin metabolite profiles across the melanoma subtypes and ICI therapy response. However, our data does suggest individual metabolites are enriched in rare melanoma subtypes. Further, the minimal variation that was observed could represent differences in polyunsaturated fatty acids or arachidonic acid metabolites. Particularly, we found that the ICI response is most influenced by polyunsaturated fatty acids. Interestingly, we repeatedly found differences in AA metabolism specifically between cutaneous and rarer melanoma subtypes, in that patients with cutaneous melanoma tended to have higher serum levels of these metabolites than patients with mucosal melanoma. However, given the little variation between our samples, more studies are needed to determine how influential these highlighted differences truly are.

We identified one oxylipin, prostaglandin J_2_ (PGJ_2_), that recurrently showed statistical differences both across melanoma subtypes and ICI responses. We found that this oxylipin is lower in cutaneous melanoma patients than in acral, mucosal, and uveal melanoma patients. When we looked at PGJ_2_ in mucosal anatomic locations, we found that this oxylipin was higher in vulvovaginal, nasopharyngeal, and anorectal than in cutaneous melanoma. Lastly, we found that CM responders had lower baseline levels of this oxylipin than mucosal responders.

PGJ_2_ and its derivative, 15-deoxy-Δ12,14-prostaglandin J_2_ (15d-PGJ_2_), have shown promise in cancer research, particularly for melanoma. Studies reveal that PGJ_2_ can inhibit cancer cell proliferation by inducing cell cycle arrest and apoptosis, primarily through activation of the peroxisome proliferator-activated receptor-gamma (PPARγ) pathway, which regulates genes that control cell growth and differentiation, making PGJ_2_ a candidate for targeting cancer cell survival mechanisms [22]. Additionally, PGJ_2_ has cytotoxic properties that enable it to disrupt proteins essential for tumor growth, which has been explored in melanoma as well as in breast, colon, and prostate cancers [23].

PGJ2 also plays a complex and context-dependent role in immunity and can modulate the tumor immune microenvironment. Through its electrophilic cyclopentenone structure and the derivative 15d-PGJ2, PGJ2 activates PPARγ and inhibits NF-κB signaling, thereby suppressing the production of pro-inflammatory cytokines and reshaping myeloid cell function [22,23,24]. These pathways have been linked to alternative macrophage polarization and changes in antigen presentation, which can either support or constrain anti-tumor T-cell responses depending on context [22,24,25]. In preclinical models, prostaglandin pathway modulation alters sensitivity to checkpoint blockade, suggesting that PGJ2-regulated networks may influence the balance between immune activation and suppression during PD-1/CTLA-4 inhibition [6,7,8,24,25,26]. Thus, our observation that circulating PGJ2 differs by melanoma subtype and ICI response raises the possibility that PGJ2 is not merely a bystander metabolite but a surrogate of broader prostaglandin-driven immune programs that could modulate immunotherapy efficacy. Prospective functional studies will be needed to define whether PGJ2 is directly targetable or primarily serves as a biomarker of prostaglandin pathway activity in melanoma.

The main limitation of our study was the small sample size. We included rare melanoma patients for whom sample collection is inherently less frequent, making it difficult to obtain large cohorts of specimens at a single institution. Because of the limited number of available samples, particularly for rare melanoma subtypes, the study was not powered to detect small or moderate effect sizes. Therefore, the findings should be interpreted as exploratory and hypothesis-generating. In the future, a multi-institutional approach would be beneficial for increasing sample numbers of rare melanoma subtype patients and ICI-treated patients to increase statistical power for our initial observation of trending differences in polyunsaturated fatty acids and metabolites in the arachidonic acid pathway. Additionally, mucosal melanoma cases were intentionally enriched in this cohort due to both the need to adequately represent distinct mucosal anatomic sites (nasopharyngeal, vulvovaginal, and anorectal) for subtype-specific oxylipin profiling. This design enabled evaluation of biologic heterogeneity across primary mucosal sites that is not typically feasible in population-representative cohorts. Finally, clinical variables such as tumor mutational burden (TMB), lactate dehydrogenase (LDH) levels, and metastatic burden were not consistently available across patients, limiting our ability to incorporate these factors into multivariate analyses.

## 5. Conclusions

Immunotherapy has significantly improved clinical outcomes for patients with late-stage melanoma, yet a substantial portion of patients fail to respond to these treatments. The variability in responses to ICI therapy, both among individual patients and across different melanoma subtypes, underscores the need to explore the influence of circulating factors such as oxylipins on therapeutic outcomes. This study highlights the intricate role of oxylipin metabolism in melanoma and its potential to affect ICI efficacy. In particular, targeting specific metabolites like PGJ_2_ may open new avenues for personalized treatment strategies, especially for patients with rare melanoma subtypes. Further research is essential to deepen our understanding of these relationships and to translate these findings into improved clinical practice.

## Figures and Tables

**Table 1:** Demographics of patients included in the study.

	Cutaneous (*n* = 6)	Acral (*n* = 7)	Mucosal (*n* = 23)			Uveal (*n* = 7)
			Nasopharyngeal (*n* = 8)	Vulvovaginal (*n* = 8)	Anorectal (*n* = 7)	
Gender, No. (%)						
Female	3 (50.0)	3 (42.9)	5 (62.5)	8 (100)	3 (42.9)	4 (57.1)
Male	3 (50.0)	4 (57.1)	3 (37.5)	0 (0)	4 (57.1)	3 (42.9)
Age (years)						
Mean ± SD	72 ± 9	60 ± 11	70 ± 15	57 ± 16	62 ± 10	57 ± 9
Range	(64–84)	(39–70)	(47–92)	(39–91)	(47–80)	(40–66)
ICI, No. (%)						
None	0 (0)	1 (14.3)	2 (25.0)	1 (12.5)	3 (42.9)	4 (57.1)
Anti-PD-1	3 (50.0)	4 (57.1)	5 (62.5)	4 (50.0)	2 (28.6)	1 (14.3)
Combination	3 (50.0)	2 (28.6)	1 (12.5)	3 (37.5)	2 (28.6)	2 (28.6)
Response, No. (%)						
No ICI	0 (0)	1 (14.3)	2 (25.0)	1 (12.5)	3 (42.9)	4 (57.1)
Non-responder	2 (33.3)	6 (85.7)	4 (50.0)	4 (50.0)	1 (14.3)	0 (0)
Responder	4 (66.7)	0 (0)	2 (25.0)	3 (37.5)	3 (42.9)	3 (42.9)

## Data Availability

The data presented in this study are available on request from the corresponding author. The data are not publicly available due to privacy or ethical restrictions.

## Supplementary Materials

The following supporting information can be downloaded at: https://www.mdpi.com/article/10.3390/metabo16010014/s1, Supplementary Table and Figures (Supplementary Table S1. Characteristics and UHPLC-MS parameters of oxylipins; Supplementary Figure S1. Diagram of oxylipin classes analyzed in this study; Supplementary Figure S2. Mass spectrometry screen for oxylipin levels in melanoma patient serum samples; Supplementary Figure S3. Comparisons with trend effects of subtype and cancer-associated oxylipins for comparison; Supplementary Figure S4. Comparisons of cancer-associated oxylipins between mucosal melanoma anatomic locations; Supplementary Figure S5. Comparisons of major prostaglandins between ICI therapy responders and non-responders for each melanoma subtype; Supplementary Figure S6. Line graphs of linoleic acid-related metabolism oxylipins for matched pre- and on-/post-treatment samples), Data S1: Shapiro–Wilk results, Data S2: Statistics for baseline serum oxylipins across melanoma subtypes, Data S3: Statistics for baseline serum oxylipins across mucosal melanoma anatomic locations, Data S4: Statistics for baseline serum oxylipins between immune checkpoint therapy responders and non-responders, Data S5: Statistics for serum oxylipins between pre-treatment and on-/post-treatment.

## Abbreviations

The following abbreviations are used in this manuscript:

AA	arachidonic acid
ACN	acetonitrile
AM	acral melanoma
CD8^+^	CD8-positive T cells
CM	cutaneous melanoma
COX	cyclooxygenases
COX-2	cyclooxygenase 2
CR	complete response
CTLA-4	cytotoxic T lymphocyte 4
CYP	cytochrome P450 enzymes
DGLA	dihomo-gamma-linolenic acid
DHA	docosahexaenoic acid
EP4	prostaglandin E2 receptor 4
EPA	eicosapentaenoic acid
HDoHE	hydroxydocosahexaenoic acids
HEPE	hydroxyeicosapentaenoic acids
HETE	hydroxyeicosatetraenoic acids
ICI	immune checkpoint inhibitor
IRB	Institutional Review Board
LA	linoleic acid
LDH	lactate dehydrogenase
LOX	lipoxygenases
LT	leukotrienes
MeOH	methanol
MM	mucosal melanoma
NR	non-responders
PC	principal component
PCA	principal component analysis
PD	progressive disease
PD-1	programmed death 1
PG	prostaglandins
PGE2	prostaglandin E2
PGJ2	prostaglandin J2
15d-PGJ2	15-deoxy-Δ12,14 prostaglandin J2
PPARγ	peroxisome proliferator-activated receptor-gamma
PR	partial response
PUFA	polyunsaturated fatty acids
R	responders
RCF	relative centrifugal force
RECIST	response evaluation criteria in solid tumors
RvD1	resolvin D1
SID	stable isotope dilution
SD	stable disease/standard deviation
TMB	tumor mutational burden
TX	thromboxanes
UHPLC-MS	ultra high-pressure liquid chromatography mass spectrometry
UM	uveal melanoma
