# Supplementary material for: Profiling Serum Oxylipin Metabolites Across Melanoma Subtypes and Immunotherapy Responders
Source: Metabolites. 2025 Dec 23;16(1):14. doi: 10.3390/metabo16010014 (PMC12844009; doi:10.3390/metabo16010014)
Supplement: Supplementary file 1 [file metabolites-16-00014-s001.zip › Supplementary Table and Figures.pdf]

Compound	Formula	Pathway	Parent	MedRt	Polarity	Standard
11β-13,14-dihydro-15-keto Prostaglandin F2α	C20H34O5	AA metabolism	353.23	3.239	[M-H]-	11β-13,14-dihydro-15-keto Prostaglandin F2α d9
12(S)-HETE	C20H32O3	AA metabolism	319.23	4.477	[M-H]-	12(S)-HETE-d8
13,14-dihydro-15-keto Prostaglandin D2	C20H32O5	AA metabolism	351.22	3.395	[M-H]-	13-14-dihydro-15-keto Prostaglandin D2-d9
13,14-dihydro-15-keto Prostaglandin F2α	C20H34O5	AA metabolism	353.23	3.297	[M-H]-	13,14-dihydro-15-keto Prostaglandin F2α/E1 d4
15-deoxy-12,14-Prostaglandin D2	C20H30O4	AA metabolism	333.21	3.889	[M-H]-	13,14-dihydro-15-keto Prostaglandin D2 d4
15-keto-Prostaglandin E2	C20H30O5	AA metabolism	349.20	3.054	[M-H]-	15-keto-Prostaglandin E2 d8
15(S)-HETE	C20H32O3	AA metabolism	319.23	4.331	[M-H]-	15(S)-HETE-d8
2,3-dinor-11β-Prostaglandin F2α	C18H30O5	AA metabolism	325.20	2.674	[M-H]-	2,3-dinor-11β-Prostaglandin F2α d4
5(S)-HETE	C20H32O3	AA metabolism	319.23	4.736	[M-H]-	5(S)-HETE-d8
6-keto-Prostaglandin F1α	C20H34O6	AA metabolism	369.23	2.588	[M-H]-	6-keto Prostaglandin F1a-d4
Leukotriene B4	C20H32O4	AA metabolism	335.22	3.726	[M-H]-	Leukotriene B4-d4
Prostaglandin A2	C20H30O4	AA metabolism	333.21	3.731	[M-H]-	Prostaglandin A2-d4
Prostaglandin D2	C20H32O5	AA metabolism	351.22	3.109	[M-H]-	Prostaglandin D2-d9
Prostaglandin E2	C20H32O5	AA metabolism	351.22	3.012	[M-H]-	Prostaglandin E2-d9
Prostaglandin J2	C20H30O4	AA metabolism	333.21	3.53	[M-H]-	Prostaglandin J2/B2 d4
17(S)-HDHA	C22H32O3	DHA metabolism	343.23	4.352	[M-H]-	17(S)-HDHA-d5
Maresin 1	C22H32O4	DHA metabolism	359.22	3.794	[M-H]-	Maresin 1 d5
Maresin 2	C22H32O4	DHA metabolism	359.22	3.945	[M-H]-	Maresin 2 d5
Protectin D1	C22H32O4	DHA metabolism	359.22	3.702	[M-H]-	Protectin D1 d5
15(S)-HEPE	C20H30O3	EPA metabolism	317.21	4.163	[M-H]-	15(S)-HEPE-d5
12(13)-DiHOME	C18H34O4	LA metabolism	313.24	4.013	[M-H]-	12(13)-DiHOME d4
13-OxoODE	C18H30O3	LA metabolism	293.21	4.31	[M-H]-	13-OxoODE-d3
13(S)-HODE	C18H32O3	LA metabolism	295.23	4.31	[M-H]-	13(S)-HODE-d4
9-OxoODE	C18H30O3	LA metabolism	293.21	4.386	[M-H]-	9-OxoODE-d3
9(10)-DiHOME	C18H34O4	LA metabolism	313.24	3.939	[M-H]-	9(10)-DiHOME d4
9(S)-HODE	C18H32O3	LA metabolism	295.23	4.379	[M-H]-	9(S)-HODE-d4
Dihomo-γ-Linolenic Acid (DGLA)	C20H34O2	LA metabolism	305.25	5.732	[M-H]-	Dihomo-y-Linolenic Acid-d6
Arachidonic Acid (AA)	C20H32O2	Polyunsatured fatty acids	303.23	5.612	[M-H]-	Arachidonic Acid-d8
Docosahexaenoic Acid (DHA)	C22H32O2	Polyunsatured fatty acids	327.23	5.509	[M-H]-	Docosahexaenoic Acid-d5
Docosapentaenoic Acid	C22H34O2	Polyunsatured fatty acids	329.25	5.658	[M-H]-	Docosapentaenoic Acid-d5
Eicosapentaenoic Acid (EPA)	C20H30O2	Polyunsatured fatty acids	301.22	5.275	[M-H]-	Eicosapentaenoic Acid-d5
Linoleic Acid (LA)	C18H32O2	Polyunsatured fatty acids	279.23	5.658	[M-H]-	Linoleic Acid-d11
α-Linolenic Acid (ALA)	C18H30O2	Polyunsatured fatty acids	277.22	5.333	[M-H]-	a-Linolenic Acid-d5

Supplementary Table S1. Characteristics and UHPLC-MS parameters of oxylipins.

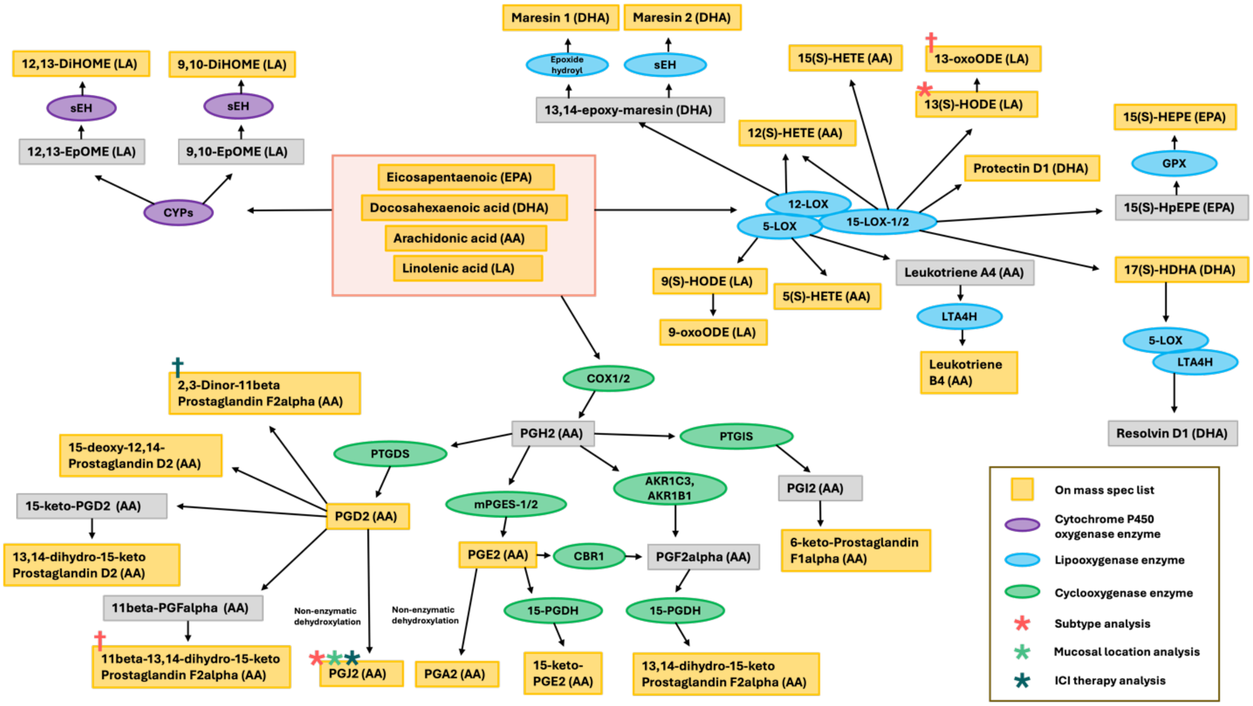

Supplementary Figure S1. Diagram of oxylipin classes analyzed in this study. Figure adapted and modified from Chistyakov and colleagues (2022). Asterisk above the listed oxylipin indicates a significant main affect in its respective analysis ($p < 0.05$). Cross above listed oxylipin indicates a trend effect in its respective analysis ($p < 0.100$).

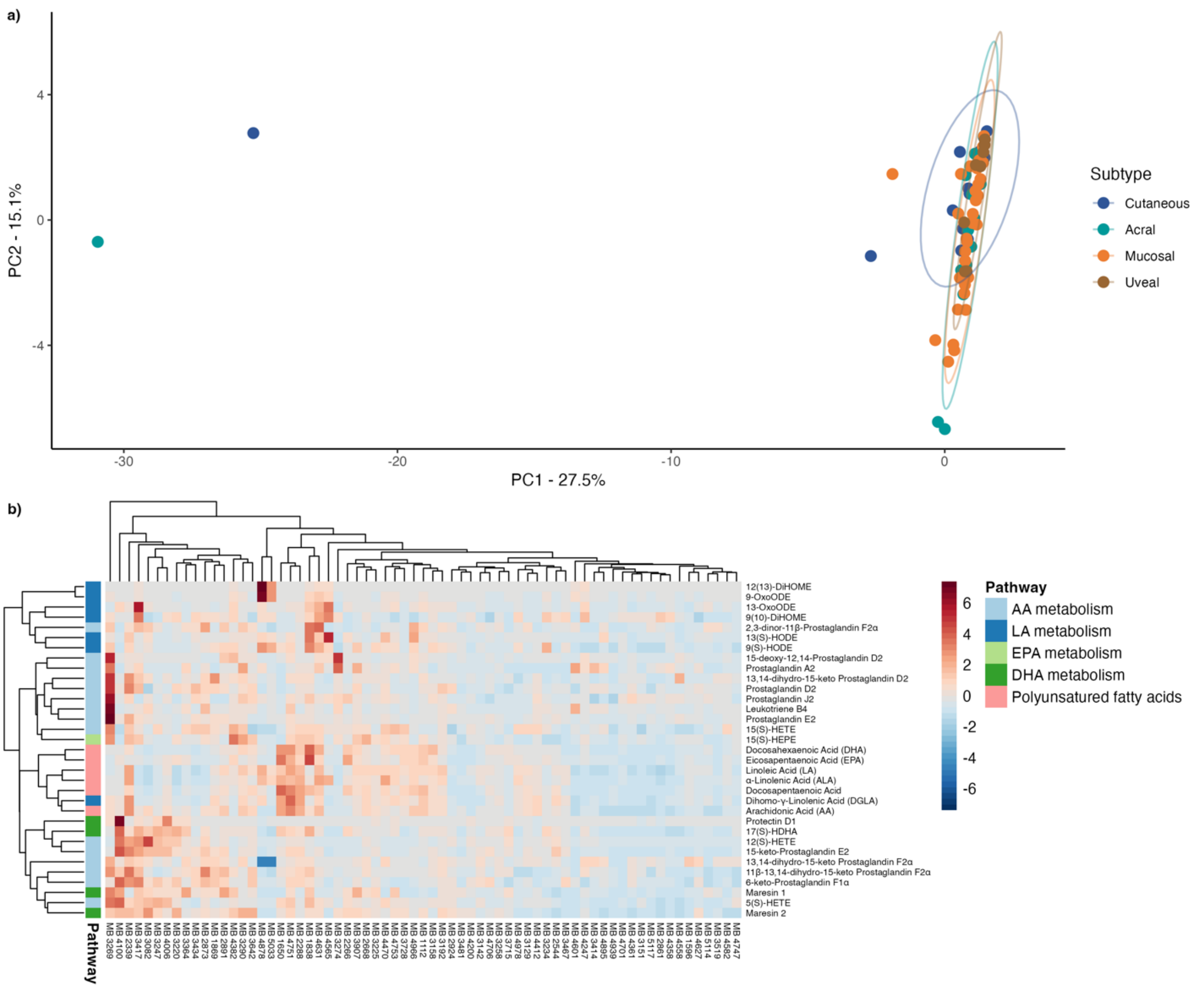

Supplementary Figure S2. Mass spectrometry screen for oxylipin levels in melanoma patient serum samples. **(a)** Principal component analysis of all samples that underwent mass spectrometry analysis. Plot demonstrates four outliers from total dataset, which were removed from further analysis. **(b)** Heatmap of all serum samples included in the study.

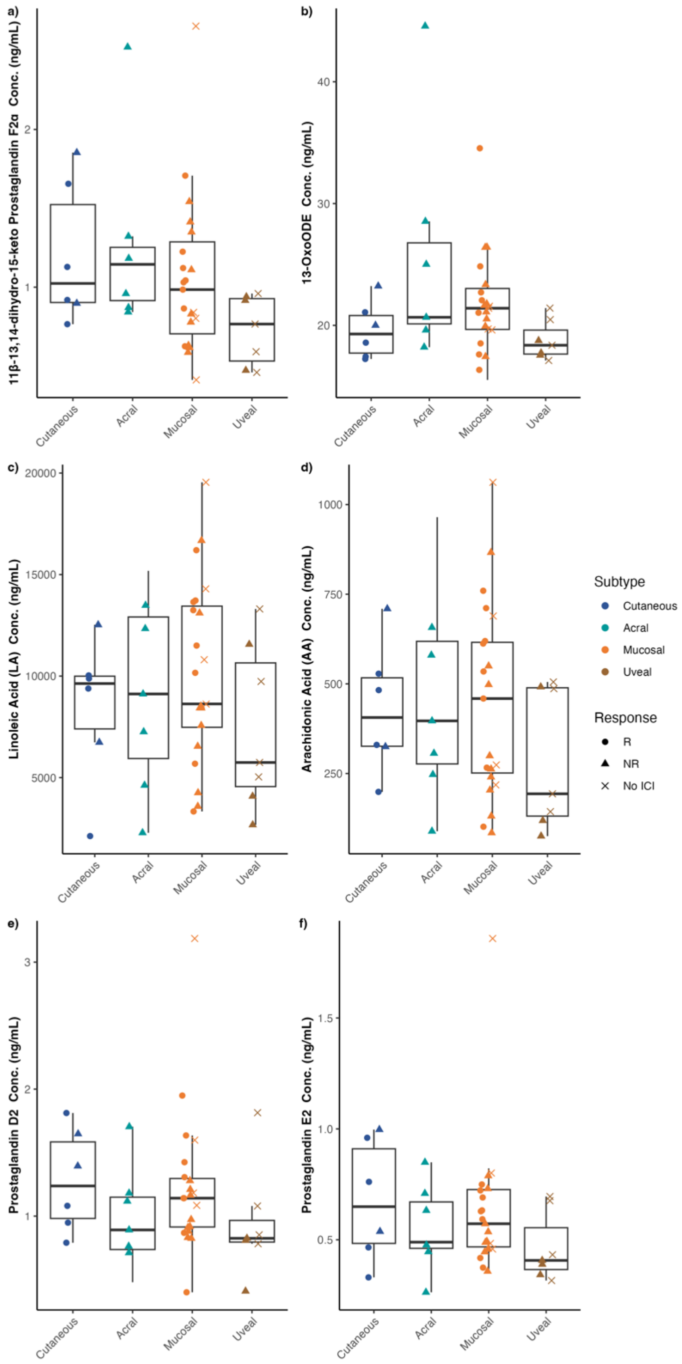

Supplementary Figure S3. Comparisons with trend effects of subtype and cancer-associated oxylipins for comparison. Boxplots of absolute serum concentration of **(a)** 11β-13,14-dihydro-15-keto Prostaglandin F2α, **(b)** 13-oxoODE, **(c)** linoleic acid, **(d)** arachidonic acid, **(e)** prostaglandin D2, and **(f)** prostaglandin E2 for all melanoma subtypes prior to any ICI therapy. Significant differences are indicated by an asterisk ($p \leq 0.05$). Trend differences are indicated by a cross ($p \leq 0.100$).

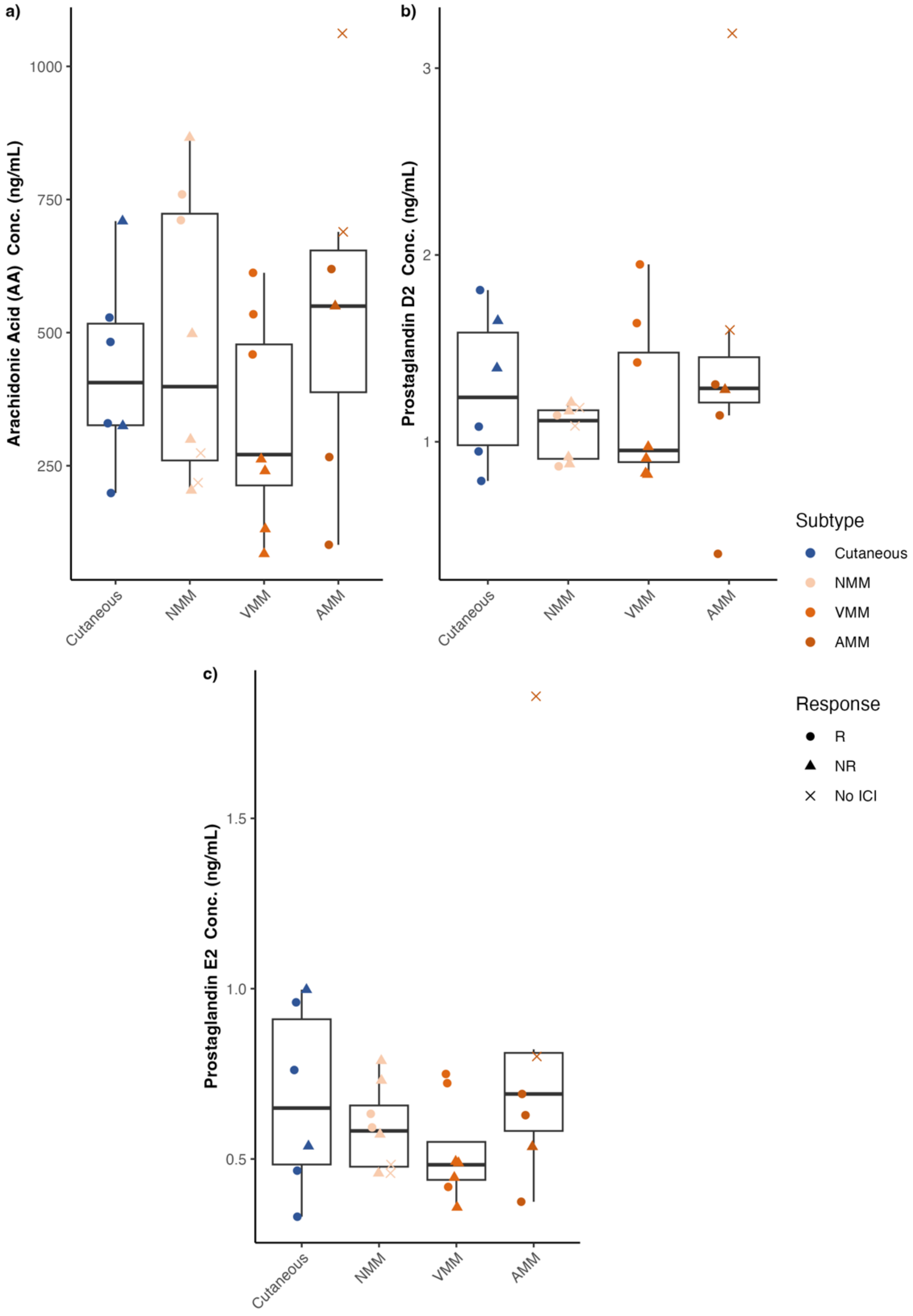

Supplementary Figure S4. Comparisons of cancer-associated oxylipins between mucosal melanoma anatomic locations. Boxplots of absolute serum concentration of **(a)** arachidonic acid, **(b)** prostaglandin D2, and **(c)** prostaglandin E2 for all mucosal melanoma locations prior to any ICI therapy. Significant differences are indicated by an asterisk ($p \leq 0.05$). Trend differences are indicated by a cross ($p \leq 0.100$).

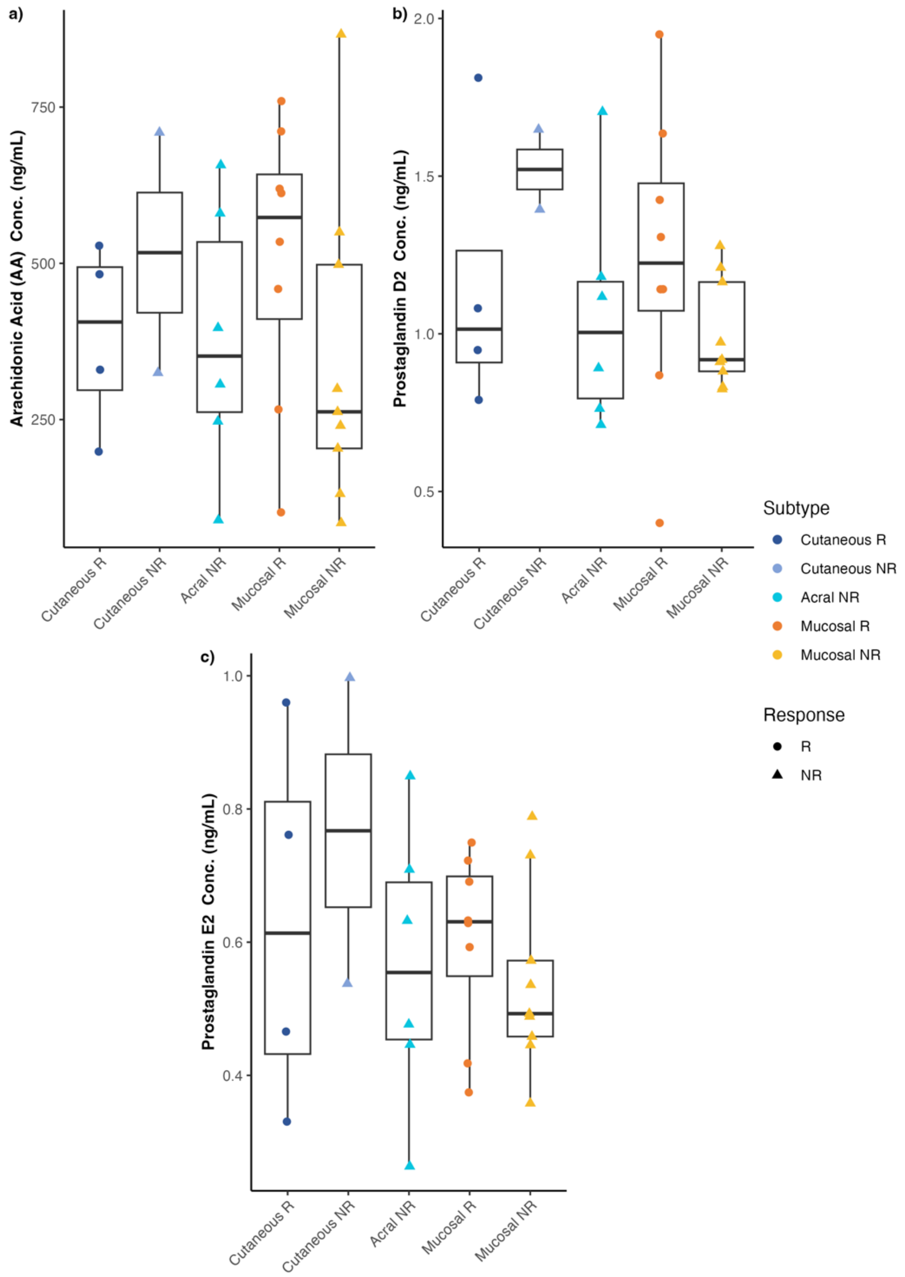

Supplementary Figure S5. Comparisons of major prostaglandins between ICI therapy responders and non-responders for each melanoma subtype. Boxplots of absolute serum concentration of **(a)** arachidonic acid, **(b)** prostaglandin D2, and **(c)** prostaglandin E2 for ICI therapy responders and non-responders for each melanoma subtype. Acral responders were not included in these plots as there were no acral responders in our study population. Significant differences are indicated by an asterisk ($p \leq 0.05$). Trend differences are indicated by a cross ($p \leq 0.100$).

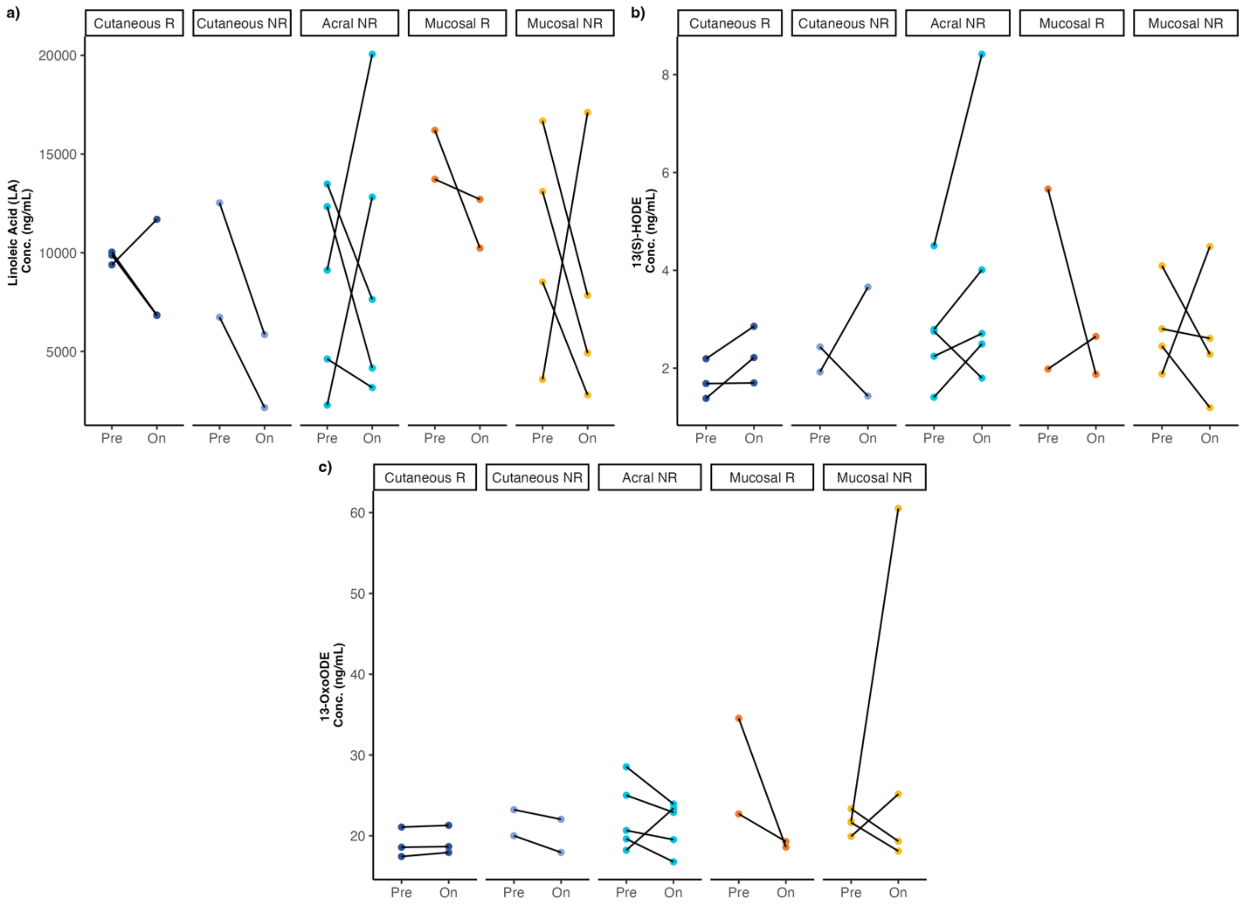

Supplementary Figure S6. Line graphs of linoleic acid-related metabolism oxylipins for matched pre- and on/post-treatment samples absolute serum concentration of **(a)** linoleic acid, **(b)** 13(S)-HODE, and **(c)** 13-OxoODE for ICI therapy responders and non-responders across melanoma subtypes prior to any ICI therapy.
